# 80 Examining the Relationship between Burn Injury Acuity and Risk for Developing PTSD and Depression Symptoms

**DOI:** 10.1093/jbcr/irad045.054

**Published:** 2023-05-15

**Authors:** Hannah Shoemaker, Yulia Gavrilova, Steven Kahn, Raleigh Cerre, Shay Gaymon, Julia Ficalora

**Affiliations:** Medical University of South Carolina, Charleston, South Carolina; The South Carolina Burn Center / Medical University of South Carolina, Charleston, South Carolina; The South Carolina Burn Center/Medical University of South Carolina, Charleston, South Carolina; Medical University of South Carolina, Charleston, South Carolina; Medical University of South Carolina, Charleston, South Carolina; Medical University of South Carolina, Charleston, South Carolina; Medical University of South Carolina, Charleston, South Carolina; The South Carolina Burn Center / Medical University of South Carolina, Charleston, South Carolina; The South Carolina Burn Center/Medical University of South Carolina, Charleston, South Carolina; Medical University of South Carolina, Charleston, South Carolina; Medical University of South Carolina, Charleston, South Carolina; Medical University of South Carolina, Charleston, South Carolina; Medical University of South Carolina, Charleston, South Carolina; The South Carolina Burn Center / Medical University of South Carolina, Charleston, South Carolina; The South Carolina Burn Center/Medical University of South Carolina, Charleston, South Carolina; Medical University of South Carolina, Charleston, South Carolina; Medical University of South Carolina, Charleston, South Carolina; Medical University of South Carolina, Charleston, South Carolina; Medical University of South Carolina, Charleston, South Carolina; The South Carolina Burn Center / Medical University of South Carolina, Charleston, South Carolina; The South Carolina Burn Center/Medical University of South Carolina, Charleston, South Carolina; Medical University of South Carolina, Charleston, South Carolina; Medical University of South Carolina, Charleston, South Carolina; Medical University of South Carolina, Charleston, South Carolina

## Abstract

**Introduction:**

Individuals who experience traumatic events are at heightened risk for developing adverse mental health outcomes, specifically posttraumatic stress and depressive symptoms. Those hospitalized following a burn injury experience significant psychological and emotional burden during acute burn treatment. Symptoms of depression and posttraumatic stress are common following a traumatic burn injury; however, little is known about the impact of burn-related intensive care unit (ICU) admission on future risk of developing posttraumatic stress disorder (PTSD) and depression. Thus, the current study examined the associations between burn injury acuity (defined by admission to ICU vs. non-ICU) and risk for developing mental health problems (i.e., depression and PTSD) among burn center patients assessed within the initial 30 days following their injury.

**Methods:**

Participants included 286 patients admitted to the burn center at a southeastern academic medical center (M_age_ = 46.79, SD = 17.47; 53% White; 66% Male; 94% Non-Hispanic; 25% admitted to ICU). Patients were assessed by the hospital’s burn behavioral health program. Risk of PTSD and depression was assessed via the Injured Trauma Survivor Screen (ITSS), a validated measure used to determine risk of PTSD and depression following an injury.

**Results:**

Independent samples t-tests revealed that ICU admission was associated with an increased risk for developing posttraumatic stress (p = .022) and depressive symptoms (p = .016) as compared to non-ICU admitted patients when assessed within the initial 30 days following a burn injury.

**Conclusions:**

Though preliminary, results highlight the importance of considering burn acuity when evaluating risk of developing psychopathology following a burn injury. Admission to ICU appears to play a key role in individuals’ risk for developing PTSD and depressive symptoms. Both physical and emotional healing are critical components of a patient’s recovery process following a burn injury.

**Applicability of Research to Practice:**

Given these results, it is important to prioritize promoting psychological recovery following a potentially traumatic burn injury in those admitted to ICU through early screening and intervention. Early intervention should emphasize reducing avoidance of feared stimuli, gradual exposure, relaxation strategies, social support, and behavioral activation techniques particularly for patients admitted to the ICU to help mitigate negative effects of trauma exposure that may lead to increased psychopathology.

